# Understanding Disease Tolerance and Resilience

**DOI:** 10.1371/journal.pbio.1002513

**Published:** 2016-07-11

**Authors:** Lauren A. Richardson

**Affiliations:** Public Library of Science, San Francisco, California, United States of America

## Abstract

Our first ever Open Highlights explores recent Open Access research into the complex relationship between host and pathogen during the course of an infection, and the factors that determine its eventual outcome.

On the surface, illness due to infection seems straightforward. An infected individual mounts an immune response, the immune system either kills or clears the pathogen, and health is restored. In reality, though, the relationship between infection and health is extremely complex. First, we have to consider disease tolerance. Within a population, some individuals are more tolerant to specific pathogens, meaning that they can be infected with larger numbers of pathogens without suffering severe illness and death. Second, there is resilience, which determines whether an individual can recover from illness. Both tolerance and resilience are dependent on host and pathogen genetics, and they complicate the path from infection back to health.

Factoring tolerance and resilience into our thinking of health has been very challenging but in two recent papers published in *PLOS Biology*, Brenda Torres, Jose Henrique Oliveira, Alexander Louie, Kyung Han Song, David Schneider and colleagues tackle these questions by developing mathematical models that are both simple and sophisticated.

In an effort to understand what differentiates more- and less-resilient individuals, Schneider and colleagues mapped how mice responded to infection with the mouse malarial parasite *Plasmodium chabaudi* in the paper “Tracking Resilience to Infections by Mapping Disease Space” [1]. The map they start with uses x,y coordinates. Imagine that the point 0,0 represents good health; as the mice get sick from the infection, they move away from the origin, but as they get better they loop back, eventually returning to the starting point. This map represents a path through “disease space” (Fig 1). The authors find that resilient mice make small loops through this space, while less resilient mice make larger loops, reflecting a longer infection time with more severe symptoms. Importantly, they demonstrate that this looping behavior applies to human disease and that these maps can be constructed from cross-sectional data gathered in field trails. The authors suggest that this approach could help distinguish resilient patients from those that will need a more aggressive course of therapy.

**Fig 1:**
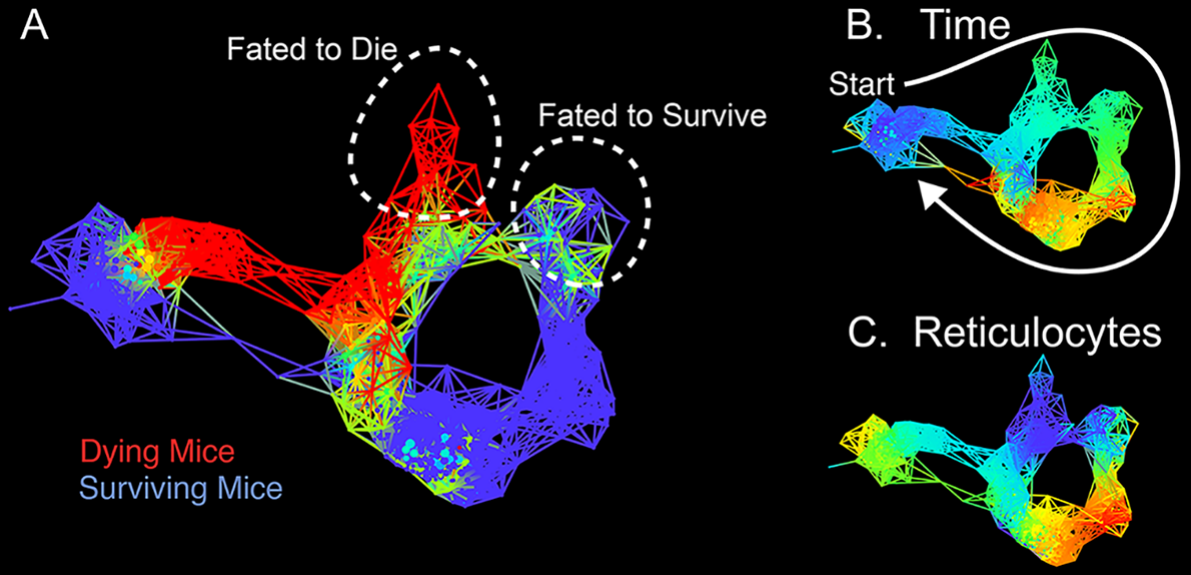
A Journey through Disease Space. Topological network maps from the Schneider lab show the paths through disease space of malaria-infected mice. In panel A, mice that succumb to infection do not show the characteristic looping path, which distinguishes them from more resilient mice. The same map is shown in panel B and C, but colored according to time or reticulocyte density. *Image credit*: *10*.*1371/journal*.*pbio*.*1002436*.*g003*.

Another wrinkle is that it is still unclear whether the damage caused by an infection is primarily due to the actions of the pathogen or due to the immune system’s efforts to control the infection. In the article “How Many Parameters Does It Take to Describe Disease Tolerance?” [2] the group develops a model to connect disease tolerance, microbe growth and the immune response using the fruit fly *Drosophila melanogaster* infected with the pathogenic bacterium *Listeria monocytogenes*. By modeling these parameters with sigmoid curves, they were able to show that–for this host and pathogen–damage was caused by the pathogen, rather than by an overzealous immune system.

In a recent paper published in *PLOS Pathogens*, Gabriela Olivera, Martin Rottenberg and colleagues uncovered a host immune response which at first glance appears to be damaging, but that on further study promotes disease tolerance [3]. *Trypanosoma brucei*, the parasite which causes African sleeping sickness, crosses the blood-brain barrier where it induces neurological dysfunction. Previous research had suggested that both *T*. *brucei* and the inflammatory response mediated by nitric oxide released in the brain caused damage. In this report though, the authors found that the nitric oxide is critical for preserving the stability of the blood-brain barrier, which is key for preventing the unlimited influx of inflammatory cells into the brain and for limiting neuroinflammation.

Jonathan Maelfait, Kenny Roose, Rudi Beyaert, Xavier Saelens, Geert van Loo, and colleagues demonstrate in another *PLOS Pathogens* paper that modulation of the immune response can increase the tolerance of mice, in this case to influenza A infection [4]. In this study, they specifically delete the gene encoding A20, a protein known to be a negative regulator of antiviral immune responses. To their surprise, loss of A20 in bronchial epithelial cells had the opposite effect to that predicted, with null mice better protected against influenza A challenge. They find that these mice have a dampened immune response, leading to more tolerant mice.

Host genetics are known to impact tolerance, but exact mechanisms are few. In a paper, also in *PLOS Pathogens*, Sarah Merkling, Ronald Van Rij, and coauthors identify an epigenetic regulator, the histone methyltransferase G9a, which mediates tolerance to RNA virus infection in *Drosophila* [5]. They find that flies lacking G9a are more sensitive to infection with an RNA virus, and die at faster rates despite an equal pathogen load as wild-type flies. They connect this with aberrant regulation of the Jak-Stat pathway in these mutant flies, a signaling pathway critical to antiviral defense. Thus epigenetic regulation of the Jak-Stat pathway is an important tolerance mechanism.

Tolerance to a pathogen can also be mediated by other disease states. Surprisingly, while influenza infection exacerbates asthma symptoms, work published in *PLOS Pathogens* by Yoichi Furuya, Dennis Metzger, and coauthors, shows that asthmatic mice are more tolerant to influenza infection than non-asthmatic mice [6]. They demonstrate that this is not due to increased immunity but instead to a strong anti-inflammatory TGF-β response triggered by asthma.

Being tolerant has more benefits than just increased health, as shown in the *PLOS ONE* paper by Sonia Altizer and colleagues [7]. By tracking the birth locations and parasite loads of monarch butterflies, they find that uninfected butterflies originated from more distant locations, indicating that they were able to migrate farther than parasitized butterflies. Interestingly, this long migration leads to a lower parasite prevalence in the population as sick, pathogen-carrying butterflies were removed from the population.

For more detailed reading please see the associated PLOS Collection [8].
